# Sleep quality in Behçet’s disease: a systematic literature review

**DOI:** 10.1007/s00296-022-05218-w

**Published:** 2022-10-04

**Authors:** N. Italiano, F. Di Cianni, D. Marinello, E. Elefante, M. Mosca, R. Talarico

**Affiliations:** Rheumatology Unit, Azienda Ospedaliero Universitaria Pisana, University of Pisa, Via Roma 67, 56126 Pisa, Italy

**Keywords:** Behçet’s syndrome, Sleep disorder, Quality of life, Rare diseases

## Abstract

Behçet’s Disease (BD) can be correlated with sleep impairment and fatigue, resulting in low quality of life (QoL); however, a comprehensive evaluation of this issue is still missing. We performed a systematic literature review (SLR) of existing evidence in literature regarding sleep quality in BD. Fifteen papers were included in the SLR. Two domains were mainly considered: global sleep characteristics (i) and the identification of specific sleep disorders (ii) in BD patients. From our analysis, it was found that patients affected by BD scored significantly higher Pittsburgh Sleep Quality Index (PSQI) compared to controls. Four papers out of 15 (27%) studied the relationship between sleep disturbance in BD and disease activity and with regards to disease activity measures, BD-Current Activity Form was adopted in all papers, followed by Behçet’s Disease Severity (BDS) score, genital ulcer severity score and oral ulcer severity score. Poor sleep quality showed a positive correlation with active disease in 3 out of 4 studies. Six papers reported significant differences between BD patients with and without sleep disturbances regarding specific disease manifestations. Notably, arthritis and genital ulcers were found to be more severe when the PSQI score increased. Our work demonstrated lower quality of sleep in BD patients when compared to the general population, both as altered sleep parameters and higher incidence of specific sleep disorders. A global clinical patient evaluation should thereby include sleep assessment through the creation and adoption of disease-specific and accessible tests.

## Introduction

Behçet’s Disease (BD) is a chronic systemic vasculitis which may affect small to medium vessels. It is characterized by a relapsing–remitting course and most frequently by muco-cutaneous, ocular and articular involvement [1].

Lifestyle and daily activities of BD patients are greatly affected by the complexity of the possible clinical profiles, resulting in a significant association with impaired quality of life (QoL) [2]. Sleep quality is an important item of QoL, and it is known to be commonly poorer in patients affected by rheumatic diseases when compared to the general population [3]. Moreover, previous studies have hypothesized a bidirectional association between sleep disorders and autoimmunity, although pathophysiological mechanisms have not been elucidated [4, 5].

Similarly to other systemic autoimmune diseases, BD can be correlated with sleep impairment and fatigue, resulting in low QoL [3]; however, a comprehensive evaluation of this issue is still missing. Therefore, we performed a systematic review of existing evidence in literature regarding sleep quality in BD.

## Methods

A systematic literature review (SLR) was conducted to analyze existing evidence about sleep quality in BD patients. The results of our systematic review were reported according to the PRISMA statement.

### Search strategy

A search was performed in MEDLINE via PubMed to identify studies published until October 2021, using the following terms [“Behcet Syndrome” AND “Sleep” OR “Sleep Medicine Specialty” OR “Sleep Phase Chronotherapy” OR “REM Sleep Parasomnias” OR “Sleep-Wake Transition Disorders” OR “Sleep Arousal Disorders” OR “Sleep Disorders, Intrinsic” OR “Sleep Paralysis” OR “REM Sleep Behavior Disorder” OR “Sleep Bruxism” OR “Sleep Apnea, Central” OR “Sleep Apnea, Obstructive” OR “Sleep Disorders, Circadian Rhythm” OR “Sleep, REM” OR “Sleep Stages” OR “Sleep Wake Disorders” OR “Sleep Deprivation” OR “Sleep Apnea Syndromes” OR “Sleep Initiation and Maintenance Disorders” OR “Sleep, Slow-Wave” OR “Sleep Latency” OR “Sleep Hygiene” OR “Dyssomnias” OR “Parasomnias” OR “Nocturnal Myoclonus Syndrome” OR “Night Terrors” OR “Nocturnal Paroxysmal Dystonia” OR “Somnambulism” OR “Polysomnography” OR “Narcolepsy” OR “Irresistible sleepiness, cataplexy and onset of sleep in desynchronized phase”] in all fields. Two independent reviewers have performed an examination of the references listed in the articles to identify further additional publications.

### Study selection/inclusion and exclusion criteria

Exclusion and inclusion criteria were established in advance. Publications were considered appropriate if they assessed sleep quality in BD patients, and if the language of publication was English. Exclusion criteria concerned case reports, animal studies, conference abstracts and review articles, articles not in English, articles not assessing sleep in BD.

### Participants/population

Studies were included if they investigated sleep quality in patients with a confirmed diagnosis of BD (according to the International Criteria for Behçet’s Disease [6] or according to a clinical diagnosis of BD). Studies were excluded if they involved patients without a confirmed diagnosis of BD.

### Data extraction

Title and abstracts of the identified articles were evaluated by two independent reviewers (N.I., F.D.C.) to rule out duplicates and check if inclusion and exclusion criteria were met. Full-text screening of the selected publications was independently performed by the two reviewers and meetings were held to agree on the final list of studies to be included. Any disagreement was solved through consensus among the two reviewers and in case of unresolved disagreements senior researchers were invited to participate in the discussion and take the final decision.

A data extraction form into a pre-designed excel sheet was developed to collect the following variables from the studies selected: author, journal, year of publication, type of study, study aim, number of patients involved in the study and their gender/age, number of healthy controls, sleep quality evaluation, tools used to evaluate sleep quality, specific sleep disorders taken into account, tools used to evaluate the specific sleep disorders, correlation with specific disease manifestations, correlation with BD activity, tools used to evaluate BD activity, main results obtained.

## Results

**Fig. 1:**
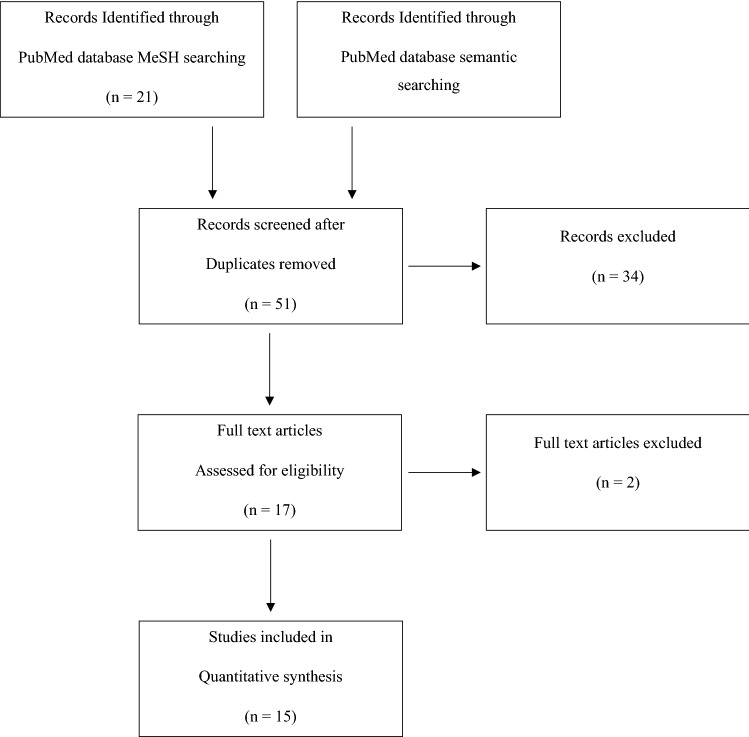
PRISMA flow diagram

As of November 22nd, 2021, 60 records were extracted by the literature research on the PubMed database (semantic: 39; MeSH 21). These records were reduced to 51 after duplicates removal and were then screened for title and abstract evaluation. Seventeen papers were eligible for a full-text evaluation, which eventually produced 15 papers to be included in our systematic review (Fig. 1).

Among the 15 papers included (Table 1), 13 were cross-sectional studies and 2 were retrospective cohort studies.

**Table 1:** Studies included in the systematic literature review

Author	Journal	Year	Type of study	Study aim	No. patients	M—F	Mean age	Healthy controls	Sleep quality evaluation	Tools to evaluate sleep quality	Specific sleep disorder evaluation	Tools to evaluate sleep disorder	Correlation with BD disease activity (tool)	Correlation with specific disease manifestations	Main results
Yeh TC et al.	Sleep and Breathing	2020	Retrospective Cohort Study	To assess the relationship between obstructive sleep apnea syndrome (OSAS) and Sjogren’s disease (Sj)/Behçet’s disease (BD)	12,962 SSj 1221 BD	80% M 20% F	39.3 in BD 54.6 in SS	51,704 non SSj 4884 non BD	No	–	OSAS	Polysomnography (PSG)	No	No	Higher prevalence of OSAS in SSj e BD: BD: HR = 1.99 [95% CI (1.06, 3.72)]; SSj: HR = 2.48 [95% CI (1.89, 3.24)]
Yazmalar et al.	International Journal of Rheumatic Diseases	2014	Cross-Sectional Study	The aim of this study was to evaluate sleep quality and the related variables in patients with BD	112	70.5% M 29.5% F	35.77 ± 8.84	67	Yes	Pittsburg Sleep Quality Index (PSQI)	No	–	No	Yes	59.8% of the patients with BD were classified as poor sleepers (total PSQI score ≥ 6). The patients with BD scored significantly higher in subjective sleep quality, sleep latency, sleep efficiency, sleep disorder, functional disorder in the morning and total PSQI compared to the healthy control subjects. There was a significant correlation between anxiety, depression, NHP and total PSQI scores. Poor sleep quality was significantly associated with genital ulcers and arthritis. No significant difference was observed between the steroids treatment modality and PSQI scores; the DMARD + colchicine group had significantly higher PSQI scores when compared with the DMARD only group
A. Gokturk et al.	Clinical and Experimental Rheumatology	2019	Cross-sectional Study	To assess the risk of OSAS in BD patients with superior vena cava syndrome (SVCS)	308 BD (28 with SVCS, 129 with vascular involvement other than SVCS, 151 non vascular)	100% M	44.3 ± 9.7 41.5 ± 8.7 40.4 ± 9.4 42.1 ± 9.4	100	No	–	OSAS	Berlin questionnaire (BQ)	No	Yes	The BQ categorised 57% of patients with SVCS (Group 1) as having a high risk for OSA, significantly higher (*p* < 0.001) compared to HCs. The frequency of those patients at high risk for OSA was 17% [OR 11.00 (95% CI 4.01–30.07)], 17 and 11% in Group 2, 3 and 4, respectively (*p* > 0.05)
Ediz et al.	The Journal of International Medical Research	2011	Cross-Sectional Study	To investigate the prevalence of restless leg syndrome (RLS), to explore potential associations with clinical features of BD, and to ascertain the prevalence of pre-existing RLS in a population with BD	109	48% M 52% F	40.4 ± 12.8	104	No	–	RLS	Epworth Sleepiness Scale (ESS)	No	Yes	The prevalence of RLS was significantly higher in patients with BD (32/109; 29.4%) than in HCs (5/104; 4.8%). No significant differences were found between BD patients with and without RLS with regard of gender, BMI and clinical features of BD. RLS severity was significantly and positively correlated only with age in BD/RLS patients (*r* = 0.56;* P* = 0.027)
A. A. Senusi et al.	Clinical and Experimental Rheumatology	2018	Cross-Sectional	To assess the association of fatigue, quality of sleep and disease activity with circulating concentration of α-MSH, VIP and inflammatory cytokines, in patients affected by BD	97	45% M 55% F	38.9 ± 1.2	30	Yes	PSQI	No	–	Yes (genital ulcer severity score (GUSS), oral ulcer severity score (OUSS) and Behçet's disease current activity form (BDCAF))	Yes	Quality of sleep pattern and daytime dysfunction items of the PSQI questionnaire had a significant impact on the fatigue score in patients with BD. The BD systemic activity, OUSS, GUSS and BDCAF were found to be more severe when the fatigue and the poor quality of sleep scores increased. Arthropathy in BD patients had a significant impact by increasing the fatigue score and amplifying the PSQI score. There was no association between any specific BD medication and the patients’ experience of either fatigue or quality of sleep scores
Tascilar et al.	J Sleep Res	2012	Cross-Sectional Study	To evaluate: sleep disorders in BD, the macrostructure of sleep in BD; the relationship of sleep disorders, PSG findings and PSG parameters with BD activity and severity; factors (primarily sleep disorders and PSG parameters) affecting fatigue; factors (primarily sleep disorders and PSG parameters) affecting quality of life (QoL)	51	39% M 61% F	40.60 ± 12.43	21	Yes	PSQI, epworth Sleepiness Scale (ESS), PSG	RLS OSAS	PSG RLS was clinically assessed	Yes (BDCAF, Behçets disease severity (BDS) score)	Yes	Insomnia was the most common complaint among patients (50.9%). 14 of 51 (27.5%) patients complained of nocturnal cramps. RLS was diagnosed in 18 patients (35.3%). EDS was found in only 7 of 51 patients (20%). OSAS was found in 13 patients (32.5%). Periodic limb movements in sleep (PLMS) with ⁄ without other sleep disorders in 11 patients (27.5%); simple snoring in 12 patients (30%); and rapid eye movement (REM) sleep without atonia (RWA) in one patient. 18 patients (45%) had neither OSAS nor RLS; only three patients (7.5%) had no sleep complaint, sleep disorder or PSG finding WASO (wake time after sleep onset), RDI (respiratory disturbance index) and AHI (apnea–hypopnea index) were significantly higher and SEI (sleep efficiency index) and SCI (sleep continuity index) were significantly lower in patients. There was no difference between the active and inactive patients regarding any of the sleep disorders (OSAS, RLS) or PSG findings
Lee et al.	Korean J Intern Med	2017	Cross-Sectional Study	To find out the effects of sleep quality in Korean patients suffering from BD	100	31% M 69% F	51 (44.5–56.0)	–	Yes	PSQI	No	–	Yes (BDCAF)	No	The group with sleep disturbance had a higher degree of pain, depression, and disease activity, with significantly lower quality of life. There were no significant differences between the good and bad sleepers with regards to CRP, ESR, drug consumption, disease duration, age, living status, and economic activity sections. Among the BDCAF subsection, the frequency of genital ulcer was significantly higher in the group with poor sleep quality. In the analysis of total PSQI and subsection PSQI, daytime dysfunction but not total PSQI was significantly higher with higher disease activity
Evcik et al.	Clinical Rheumatology	2012	Cross-Sectional Study	To investigate the association of BD with neuropathic pain syndrome (NPS) and impaired quality of life and sleep quality	111	44% M 56% F	38.49 ± 11.01	52	Yes	PSQI	No	–	No	No	The NPS was found in a ratio of 19.8% in BD (13 females and 9 males) and none in healthy controls. Also, significant increase in LANSS (Leeds Assessment of Neuropathic Symptoms and Signs) index scores was observed in patients with BD compared to healthy controls. The total LANSS score showed significant positive correlation with PSQI score in patients with BD Most of the patients with BD had poor sleep quality (69.4%) compared to healthy controls (55.8%). However, this was not statistically significant
Uygunoğlu et al.	European Neurology	2014	Cross-Sectional Study	To investigate the microstructure of sleep in patients with BD and neuroBehçet	30	BD 29.4% M 70.6% F NBD 76.5% M 23.5% F	BD: 40.2 ± 9.5 NBD: 36.1 ± 7.9	44	Yes	PSG ESS PSQI	OSAS RLS	PSG—respiratory disturbance index (RDI, the sum of apneas, hypopneas and respiratory effort-related arousals/TST), and periodic leg movement (PLM) index (number of PLM/TST)	No	No	None of the PSG parameters displayed significant differences between patients with BS and NBS. The comparison of the PSG parameters in all BD patients with the control group showed that sleep onset was significantly longer, the duration of superficial NREM sleep stage was significantly longer and RDI (Respiratory Disturbance Index) was significantly higher in patients. The PSQI and the ESS were not different between the two groups of patients, or between patients and controls. On the other hand, the presence of RLS was significantly more common in patients with BS and NBS compared to healthy subjects
Koca et al.	Clinical Rheumatology	2015	Cross-Sectional Study	To investigate the relationship between disease activity and depression and sleep quality in BD	40	72% M 28% F	18–65 years	30	Yes	PSQI	No	–	Yes (BDCAF)	No	In the BD and control groups, the sleep quality scores were 6.42 ± 4.44 and 3.12 ± 2.15 (cutoff point = 5), respectively. When the BD and control groups were compared in terms of depression and sleep quality, it was determined that the scores of both parameters were significantly higher in the BD group (*p* = 0.012 and *p* = 0.020, respectively). In the BD group, a significant positive correlation was determined between the BDCAF scores and depression and sleep quality scores (*r* = 0.559, *p* < 0.001 and *r* = 0.462, *p* = 0.003, respectively)
Ayar et al.	Modern Rheumatology	2020	Cross-Sectional Study	To compare the level of central sensitization (CS) using CS-index in patients with BD and healthy controls (HCs), and to evaluate the relationship of CS with health profile, including neuropathic pain and sleep quality in patients with BD	88	37% M 63% F	38.84 ± 9.45	60	Yes	PSQI	No	–	No	No	Sleep quality was found to be poor (PSQI score ≥ 5) in 73.6% of patients with BD and 43.3% of HCs, and the difference between the groups was significant. There was also a significant correlation between the modified CSI (Central Sensitization Index) score for PSQI and the PSQI score
Masoumi et al.	BMC Rheumatology	2020	Cross-Sectional Study	Assessment of the association of various lifestyle factors, including sleep quality, and wellness and health status in patients with BD	52	59% M 41% F	44	–	Yes	Mini-sleep questionnaire (MSQ)	No	–	No	No	Sleep quality emerged as the single most important factor associated with self-rated wellness and health status in patients with BD. Among 10 sleep quality questions, patients showed higher disruptions in five domains of sleep quality as follows: difficulty falling asleep, waking up too early, snoring, excessive daytime sleepiness and feeling tired upon waking up in the morning. Frequency distribution of each of these five domains, happening for more than three nights in a usual week, were 33, 33, 29, 29 and 27%, respectively
Toprak et al.	Turkish Society of Physical Medicine and Rehabilitation	2016	Cross-Sectional Study	To investigate the scores of depression, anxiety, sleep and QoL, to identify the prevalence of fibromyalgia (FM) in BD, and to evaluate whether there is a difference between BD patients with and without FM in these scores	97	56% M 44% F	76.3% 20–45 years 11.3% < 20yrs 12.4% > 45years	95	Yes	PSQI	No	–	No	No	There was no significant difference in the PSQI scores between BD patient and healthy control groups. The patients with both BD and FM scored significantly higher in the BDI (*p *< 0.017) and PSQI (*p* = 0.001), and scored significantly lower in the SF-36 components, except general health (*p* = 0.114), compared to the patients with only BD
Önalan et al.	Archives of Neuropsychiatry	2017	Cross-Sectional Study	To investigate the prevalence and characteristics of RLS in patients with BD and multiple sclerosis (MS)	96 BD (41 of whom NBD) 97 MS	53% M 47% F	BD: 39.9 ± 11.8 MS: 34.97 ± 4.1	100	No	–	Restless legs syndrome	RLS identification form IRLSSG rating scales	No	Yes	RLS had the most common incidence in MS group (22.7%), followed by NBD (22%), and by BD (15.6%). The incidence of RLS was significantly higher in MS and NBD groups (9 patients, 22%) compared to the control group (10 subjects, 10%) (*p *= 0.004, 0.029 respectively). In other BD, the incidence of RLS was similar to one in the control group (10.9%). No significant correlation was found between the age, gender, familial history of RLS, kinship between the parents, presence of an occupation that requires use of legs/feet and RLS. The severity score of RLS was the highest in MS group (22.18 ± 6.38), however it didn’t reach to a statistical significance (BD group 20.7 ± 5.19, control group 19.8 ± 6.97). The sleep disorder was more common in MS group, who also reported longer time to fall asleep
Chen et al.	Sleep	2016	Retrospective Cohort Study	To explore the relationship between untreated OSAS/managed OSAS and risk of autoimmune diseases, including BD	105,846 points with OSAS	76.48% M 23.52% F	47.26	423,384	No	–	OSAS	PSG	No	No	The overall cumulative incidence of autoimmune diseases in the entire OSA cohort was significantly higher than that in the matched control cohort, with an aHR of 1.94 (95% CI 1.66–2.27, *p* < 0.001). The incidence rate of RA (aHR 1.33, 95% CI 1.03–1.72, *p* < 0.01), Sjögren disease (aHR 3.54, 95% CI 2.75–4.56, *p* < 0.001) and Behçet disease (aHR 5.33, 95% CI 2.25–12.66, *p* < 0.001) were significantly higher in the OSA cohort than in the non-OSA control cohort. The overall risk of the development of autoimmune disease in patients treated for OSA was significantly lower than in patients with OSA who did not receive treatment [HR 0.51 (0.28–0.92); *p* < 0.05]

Overall, data about 121.415 BD patients were collected (84.603 males (69.7%), 36.812 females (30.3%)) with a range of age of between 18 and 65 years. No studies included patients under the age of 18.

The two most explored domains were the overall quality of sleep and the specific sleep disorders in BD, evaluated by administering one or more sleep-related non-disease-specific questionnaires and/or by performing a polysomnography (PSG).

More specifically, 8 papers [1, 6–12] out of 15 (53%) exclusively assessed global sleep quality and sleep characteristics in BD patients, by adopting the Pittsburgh Sleep Quality Index (PSQI) questionnaire in 7 [1, 6–10, 12] out of 8 papers (87, 5%) and the Mini Sleep Questionnaire (MSQ) in 1 [11] (12, 5%). On the other hand, 5 [5, 13–16] papers out of 15 (33%) exclusively evaluated the frequency of a specific sleep disorder in BD, that is Restless Leg Syndrome (RLS) in 2/5 papers [14, 15] and Obstructive Sleep Apnea Syndrome (OSAS) in 3/5 [5, 13, 16] papers. The Berlin Questionnaire (BQ) [13], the Epworth Sleepiness Scale (ESS) [14], RLS identification-form and the International Restless Legs Syndrome Study Group-Rating Scale (IRLSSG-RS) [15] were performed by one paper each, respectively to identify the risk of OSAS in a BD cohort, to examine subjective sleep quality in BD patients suffering from RLS, to make RLS diagnosis and to evaluate RLS severity. On the other hand, PSG was performed in 2 works [5, 16] out of 5 (40%) to explore OSAS. Finally, two papers [3, 17] (13%) investigated both the global sleep characteristics in a BD cohort along with the evaluation of the presence of RLS and OSAS, overall employing PSQI, ESS and PSG.

From our analysis it was found that patients affected by BD scored significantly higher PSQI compared to controls, with medium scores of PSQI ranging from 6.42 to 9.4 (value obtained from the ranges of PSQI score available in nine papers). In addition, Yazmalar et al. [1] and Tascilar et al. [3] analyzed each PSQI item, and the score of subjective sleep quality, sleep latency, sleep efficiency, sleep disorder and daily dysfunction overall appeared significantly worse in BD cohorts compared to controls. Regarding the PSG-based sleep quality evaluation, longer sleep onset time, longer nREM stages, longer wakefulness after sleep onset (WASO) and increased respiratory disturbance index (RDI) were the most often detected objective sleep alterations in BD patients.

Four papers out of 15 (27%) studied the relationship between sleep disturbance in BD and disease activity [3, 6–8]. With regards to disease activity measures, Behçet’s Disease Current Activity Form (BDCAF) was adopted in all papers, followed by Behçet’s Disease Severity (BDS) score [3], genital ulcer severity score (GUSS) and oral ulcer severity score (OUSS) [6]. Poor sleep quality showed a positive correlation with active disease in 3 out of 4 studies [6, 7, 9], whereas Tascilar et al. [3] observed no difference between active and inactive patients in terms of any sleep disorders and PSG alterations. On the other hand, in the analysis of total PSQI and subsection PSQI by Lee et al. [8], only daytime dysfunction was significantly higher in patients with higher BDCAF score.

Furthermore, six papers [1, 3, 6, 13–15] reported significant differences between BD patients with and without sleep disturbances with regard to specific disease manifestations. Notably, arthritis and genital ulcers were found to be more severe when the PSQI score increased [6]. Also, RLS and OSAS respectively showed higher incidence in BD patients with central nervous system (CNS) involvement [17] and in BD patients with superior vena cava syndrome (SVCS) [13]. Tascilar et al. [3] evaluated the polysomnographic macrostructure of sleep according to BDCAF subsections, reporting that genital ulcerations increased WASO and that oculopathy index was negatively correlated with total sleep time (TST) and time in bed (TIB). Only one study [14] found no differences between BD patients and controls with regard to disease manifestations.

No studies comparing frequency of sleep disturbances among cohorts of patients affected by BD and other autoimmune diseases were found. Nonetheless, in the study by Yeh et al. [5] incidence of OSAS was also measured in a population of patients affected by Sjogren’s syndrome (SS), showing higher rates of OSAS when compared to healthy controls (HCs).

## Discussion

Quality of sleep is known to be impaired in patients affected by systemic autoimmune diseases [3], but a systematic and comprehensive evidence was not yet available about the correlation between sleep disturbances and BD.

In our systematic review, two domains were mainly considered: global sleep characteristics (i) and the identification of specific sleep disorders (ii) in patients affected by BD.

(i) Global Sleep Assessment. Regarding the overall assessment of sleep characteristics in BD, two studies measured quantitative sleep parameters performing polysomnographic evaluation. In comparison with HCs, PSG revealed a longer sleep onset time, longer nREM stages, longer WASO, increased RDI, and significantly lower SEI (sleep efficiency index) and SCI (sleep continuity index). These results seem consistent with the difficulty in falling asleep, short sleep, lessened sleep efficiency, day-time sleepiness and fatigue, complained by BD patients in real life. Sleep features were also assessed by sleep-related non-disease-specific questionnaires. The most frequently adopted questionnaire in the publications included in our systematic review was PSQI. In almost all the studies reviewed, BD patients scored statistically significant higher PSQI values compared to HCs. One exception was the study by Toprak et al. [13], in which the statistical significance for PSQI was achieved only for the cohort of patients with both BD and fibromyalgia (FM) diagnoses. In fact, FM is a condition causing chronic widespread pain and diffuse tenderness, and it is associated to impaired neural signaling causing hyperalgesia and allodynia [18], contributing to sleep disturbances. Moreover, BD also shows frequent neurological involvement with consequent higher rates of central sensitization (CS) and neuropathic pain syndrome (NPS), which correlate with higher PSQI scores as described in the studies by Evcik et al. [9] and Ayar et al. [11]. Therefore, also considering that the incidence of FM may reach high rates in BD population [19, 20], the single contribution of such concomitant clinical conditions in inducing impaired sleep quality in BD patients still remains arguable.

(ii) Sleep disorders. In the articles included in the present work, the only two specific sleep disorders taken into consideration were OSAS and RLS. OSAS is a common condition in autoimmune diseases, as previously reported in several studies [16, 21]. Our review observed comparable data also in BD. Four studies [3, 5, 16, 17] reported statistically significant higher rates of OSAS in BD patients compared to HC, and this correlation was interpreted as a result of the chronic inflammatory state induced by the disease involvement of upper airways. Intriguingly, in the study by Chen et al. [17] it was suggested that conversely OSAS may be a risk factor for the development of autoimmune diseases including BD. According to that, primitive OSAS can contribute to systemic inflammation through the stimulation of circulating proinflammatory cytokines, chemokines, and adhesion molecules. This observation supports the presence of a bidirectional relationship between OSAS and BD, based on the existing evidence of elevated levels of various serum inflammatory cytokines in both conditions. Some evidence also suggests that higher circulating rates of inflammatory cytokines interfere with neuroendocrine activity and central nervous transmission, clarifying sleep impairment and neurological disorders in inflammatory conditions [9]. RLS was analyzed by four studies [3, 14, 15, 17] included in the present review, which unanimously showed significantly higher incidence of RLS (between 15 and 30%) in BD patients when compared to HCs. These studies disagree on the role played by disease involvement and severity in the occurrence of RLS. In fact, Önalan et al. [16] found a statistically significant difference in RLS incidence between NBD and non-NBD patients, indicating a possible connection with neurological involvement. The other studies observed no difference with regard to the clinical features of BD. Ediz et al. [15] and Tascilar et al. [3] respectively proposed iron-deficiency due to gastrointestinal inflammation and chronic pain secondary to CNS dopaminergic system dysfunction as possible hypotheses of the physio-pathological relationship between BD and RLS.

Future perspectives/unmet needs. Our review points out the impact and high frequency of sleep impairment in BD patients. Poor sleep quality definitely represents an additional impairment in BD patients and in their quality of life, which is already impacted by several factors such as sexuality [23], psychological burden [24, 25]. Consequently, patients’ examination should also include the assessment of sleep quality, usually performed through the administration of questionnaires. In the studies we included, five different questionnaires were adopted. These sleep-related questionnaires are not specific for BD nor validated for it, so they might not provide a comprehensive and accurate overview on sleep features in such patients. Secondarily, these tools differ in structure and scoring system, making results not comparable. Hence, a BD-specific questionnaire would be desirable. In addition to the tools based on the patient’s point of view, sleep characteristics should also be evaluated by objective instrumental tests. PSG examines quantitative and comparable parameters of sleep, but it is burdened by scarce feasibility and reliability since it is conducted in a non-domestic environment. An instrumental tool to perform the monitoring of sleep in real-life conditions should thus be preferable. One last point highlighted by our review is the discrepancy of the results about the correlation between sleep quality and disease activity and involvement. For a better understanding of this issue, prospective longitudinal studies accounting sleep assessment as part of the patient follow-up are required, with the aim of examining sleep features in different phases of disease. Results from these studies might offer an interesting starting point to observe whether and how sleep alterations respond to BD therapy and empower patients in addressing quality of sleep during the consultations with their specialist [26] as part of their healthcare decision-making process.

## Conclusions

The present review of the literature demonstrates lower quality of sleep in BD patients when compared to the general population, both as altered sleep parameters and higher incidence of specific sleep disorders. A global clinical patient evaluation should thereby include sleep assessment through the creation and adoption of disease-specific and accessible tests.

Future prospective studies on BD patients cohorts should be performed to investigate the correlation between disease activity and sleep quality during follow-up, also with the aim of assessing quality of life in BD by adopting a more comprehensive and multi-dimensional approach that can definitely also contribute to improve the clinical and therapeutic management.
